# Population risk factor estimates for abdominal aortic aneurysm from electronic medical records: a case control study

**DOI:** 10.1186/1471-2261-14-174

**Published:** 2014-12-04

**Authors:** Diane T Smelser, Gerard Tromp, James R Elmore, Helena Kuivaniemi, David P Franklin, H Lester Kirchner, David J Carey

**Affiliations:** Sigfried and Janet Weis Center for Research, Geisinger Health System, Danville, PA USA; Department of Vascular and Endovascular Surgery, Geisinger Health System, Danville, PA USA; Henry Hood Center for Health Research, Geisinger Health System, Danville, PA USA

**Keywords:** Aortic Aneurysm, Abdominal, Electronic medical record, Neoplasms, Benign, Risk factors, Blood pressure, Diabetes mellitus, Type 2, Case–control studies

## Abstract

**Background:**

Using abdominal aortic aneurysm (AAA) as a model, this case–control study used electronic medical record (EMR) data to assess known risk factors and identify new associations.

**Methods:**

The study population consisted of cases with AAA (*n* =888) and controls (*n* =10,523) from the Geisinger Health System EMR in Central and Northeastern Pennsylvania. We extracted all clinical and diagnostic data for these patients from January 2004 to December 2009 from the EMR. From this sample set, bootstrap replication procedures were used to randomly generate 2,500 iterations of data sets, each with 500 cases and 2000 controls. Estimates of risk factor effect sizes were obtained by stepwise logistic regression followed by bootstrap aggregation. Variables were ranked using the number of inclusions in iterations and *P* values.

**Results:**

The benign neoplasm diagnosis was negatively associated with AAA, a novel finding. Similarly, type 2 diabetes, diastolic blood pressure, weight and myelogenous neoplasms were negatively associated with AAA. Peripheral artery disease, smoking, age, coronary stenosis, systolic blood pressure, age, height, male sex, pulmonary disease and hypertension were associated with an increased risk for AAA.

**Conclusions:**

This study utilized EMR data, retrospectively, for risk factor assessment of a complex disease. Known risk factors for AAA were replicated in magnitude and direction. A novel negative association of benign neoplasms was identified. EMRs allow researchers to rapidly and inexpensively use clinical data to expand cohort size and derive better risk estimates for AAA as well as other complex diseases.

## Background

Epidemiological research studies on risk factors are traditionally performed with case–control or cohort studies, requiring a considerable sample size, cost and time investment. Electronic medical records (EMR) contain a wealth of phenotypic information with high potential to replace costly traditional epidemiological methods for purposes such as determining disease risk factors. In this study we utilized an extensive EMR to determine risk factors associated with incident cases of abdominal aortic aneurysm (AAA) in a population-based case–control study from the Geisinger Health System (GHS) serving populations in Central and Northeastern Pennsylvania [1, 2]. Pennsylvania has one of the highest rates of mortality from AAA in the USA [3].

AAA is defined as a dilatation of >3 cm in the infrarenal aorta [4–7]. A leading cause of death in the United States, AAAs often exist undetected until the aneurysm ruptures, with a concomitant fatality rate of up to 90% [5, 8–11]. Rupture can be prevented by endovascular repair or traditional open aortic surgery, which is usually performed after the aneurysm reaches a diameter of ≥5.5 cm. Since AAAs can be detected non-invasively by ultrasonography and can be surgically repaired, AAA is an ideal disease for a screening program. Ultrasonographic screening is noninvasive, relatively inexpensive and has a sensitivity and specificity of ≥99% [9, 12–14]. Currently, the United States Preventative Services Task Force (USPSTF) recommends one AAA ultrasound screening for males, 65 to 75 years of age who smoked ≥100 cigarettes in their lifetime [15, 16]. The USPSTF does not recommend screening for women, although females with AAA have a poorer prognosis and higher mortality rate in the event of a rupture [17–23]. Medicare began covering ultrasonography screening in 2007 for the initial “Welcome to Medicare” enrollment examination for men 65 to 75 years of age who have ever smoked or those with a family history of AAA [24]. According to recent studies fewer than 30% of eligible patients are actually screened [16, 25]. The current guidelines are under-utilized and exclude many at increased risk [26]. Additionally, a recent retrospective analysis indicated that 77% of ruptured AAA patients were unaware of their AAA prior to the rupture, despite a visit to a clinician within the past 5 years [27].

Current risk prediction based only on sex, age and smoking has low sensitivity and specificity, and therefore is used relatively infrequently. Better risk estimates and risk prediction models should improve utility and utilization. Our study established the feasibility of utilizing an EMR to identify novel risk factors and replicate risk factor associations with the incidence of AAA found in the literature. It also demonstrated the utility of EMRs to rapidly expand the available cohort size for identifying risk factors and obtaining refined effect size estimates.

## Methods

### Study population

GHS provides primary and specialty care to a highly stable population of 2.6 million residents in Central and Northeastern Pennsylvania [2]. Geisinger serves a large catchment area. We restricted the region to those regional divisions (counties) where Geisinger serves more than 10% of the county population. Among these counties Geisinger serves about half of their two million inhabitants. The cases and controls for this study consisted of individuals with AAA and individuals without diagnosed AAA from GHS with clinical records in the EMR from January 2004 to December 2009. Cases (*n* =888; 686 males and 202 females) were identified by the following criteria: International Classification of Diseases, 9^th^ Revision (ICD-9) codes for AAA (441.3, 441.4), Current Procedural Terminology (CPT) codes (34800, 34802, 34803, 34804, 34805, 34830, 34831, 34832, 35081, 35082, 35091, 35092, 35102, 35103, 35131, 35132), or referral to the GHS Department of Vascular Surgery with an imaging study (such as ultrasound, computed tomography scan or MRI) to confirm an infrarenal aortic diameter >3.0 cm. All AAA cases in GHS are referred to the Department of Vascular Surgery, which diminishes possible ascertainment bias.

Statistical analyses were performed using bootstrap aggregation (see Statistical Analyses section below for details). A control sample was selected to reflect population census demographics from all available patients without known AAA at the start of the study (*n* =10,523; 4,132 males and 6,391 females) enrolled in the GHS MyCode® biobanking repository [1]. All individuals in the study were Caucasian, which reflects the homogeneous ethnicity of the population in the GHS service area and the demographics of the disease. The MyCode® repository consists of individuals attending primary care sites in the communities served by GHS. Inclusion criteria were: adults >18 years of age, patient at a GHS primary care clinic, and no diagnosis of dementia. The MyCode® participants are representative of the demographic and clinical characteristics of the GHS outpatient population. Individuals gave written informed consent to allow their EMR data to be used for research purposes and to have biological specimens stored in the biobank.

GHS has utilized EMR since 1996, and implemented a data warehouse system for research data mining and analysis in 2008 [1]. This data warehouse includes the outpatient records of the patients seen by primary care and specialty providers. Analysts in the biostatistical core extract and de-identify the data through a data broker system before the investigators receive the dataset. The study was approved by the Institutional Review Board of GHS.

### Data source

Demographic and clinical variables of interest were extracted from the Geisinger EMR. Clinical risk factors were selected from the literature or were those of biological interest based on AAA pathobiology [7–9, 11, 28–42]. All diagnoses, laboratory measures and clinical values from primary care and specialty clinic visits (as of the date of the data extraction) were extracted. Age was defined as the age at AAA diagnosis for cases, and age at data extraction for the controls. Individuals >89 years of age were removed to protect potential identification of subjects. The ICD-9 codes and diagnoses used to define these variables are listed in Table 1. Since there were a number of infrequent diagnoses among the 565 distinct ICD-9 codes used for the data extraction, the codes were collapsed into 17 categories to reduce the number of variables for modeling.

**Table 1:** **ICD-9 diagnostic codes and assigned categories used to identify comorbidities**

Diagnostic category	ICD-9 codes
Arterial dissection	443.21–443.29
Atherosclerosis	440
Benign neoplasm	210.00–229.9, 238.8, 238.9
Cerebral thrombosis	434, 434.01, 434.1, 437.1
Cerebrovascular disease	434.11, 434.91, 435.8, 435.9, 436, 437, 437.0A, 437.4, 437.6–437.9
Coronary stenosis	411.81, 414.00–414.9
Cranial artery stenosis	433–433.9, 434.9, 435–435.3
Hypertension	401.0–405.99
Intracranial aneurysm	430, 437.3
Intracranial hemorrhage	431, 432.1, 432.9
Kidney disease	585.3–585.9, 586
Malignant neoplasm	140.00–209.6, 230.00–234.9, 235.1–238.3
Myelogenous neoplasm	200–208.9, 238.4–238.7, 238.71–238.73, 238.75, 238.76, 238.79, 272.2
Peripheral artery disease	440.21, 440.22, 440.3, 440.31, 440.32, 443.9
Pulmonary disease	491.00–492.8, 493.2, 496, 518.1
Type 1 diabetes	250.01, 250.03, 250.11, 250.13, 250.2, 250.23, 250.41, 250.43, 250.51, 250.53, 250.61, 250.63, 250.71, 250.73, 250.81, 250.83, 250.93
Type 2 diabetes	250.00, 250.02, 250.1, 250.12, 250.22, 250.4, 250.42, 250.5, 250.52, 250.6, 250.62, 250.7, 250.72, 250.8, 250.82, 250.9, 250.92

ICD-9, International Classification of Diseases (9^th^ Revision).

All variables were examined for consistency and distribution. Extreme, clinically or biologically implausible values were attributed to data entry error and excluded from the analysis. The median was used as a measure of centrality for continuous variables of the cleaned data set.

### Statistical analyses

**Figure 1:**
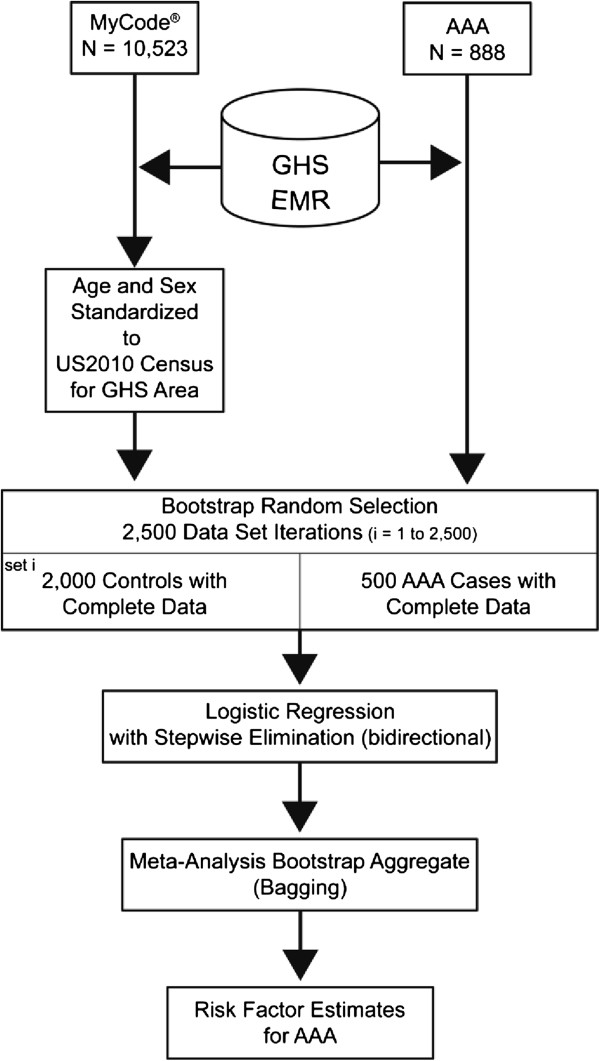
**Study design.** MyCode® is the biobank at GHS, Geisinger Health System. AAA, abdominal aortic aneurysm; EMR, electronic medical record.

U.S. Census Bureau data for 2010 [43] for all counties within the GHS service area were used to standardize the control sample for population age and sex. Traditional bootstrap methods [44, 45], with replacement, were used to randomly generate 2,500 iterations of data sets of 2,000 controls and 500 AAA cases with complete data (Figure 1). Each set of controls was selected to reflect the census age and sex demographic structure. Younger individuals, especially males, are underrepresented among patients in health care systems. To prevent oversampling of individuals under 35 years of age, census age classes (18,35] were collapsed into a single class. The number of controls was limited to 2,000 to ensure that the sampling of young males was not extreme. Cases were selected at random from the 888 available cases. The 2,500 bootstrap data sets were analyzed using logistic regression with AAA as the outcome variable and 26 explanatory variables. Variable selection was achieved by bidirectional stepwise elimination using Aikaike’s information criterion (AIC) [46] to evaluate model fit. A final model was generated using variables that were consistently retained in most bootstrap iterations. A second set of 2,500 bootstrap data sets were generated and analyzed using logistic regression with the final model of a fixed number of variables (Table 2), i.e., each bootstrap set was analyzed with the same model [47]. Regression estimates were recorded for each iteration and the estimates aggregated using meta-analytic techniques (using random-effects weighting). Variables were ranked by how often they were retained in the model, and by the *P* value, which was based on the mean z score weighted by the number of iterations the corresponding variable was included in the model. The 14 highest ranked variables were then fixed in a second bootstrap analysis (no stepwise elimination). We considered variables statistically significant at *P* <0.05, two-sided. Analyses were performed in R v.2.16.2, 64-bit (R Foundation for Statistical Computing; http://www.R-project.org, Vienna, Austria) [48] using the glm and rmeta packages.

**Table 2:** **Risk factor estimates for AAA**

Variable	Meta^*^estimate	Meta^*^SE	***P*** ^†^	OR	95% CI
Peripheral artery disease	1.49	0.0040	3.71E-16	4.42	3.08–6.35
Smoking, ever/never	1.36	0.0036	3.92E-15	3.91	2.77–5.53
Coronary stenosis	1.07	0.0032	2.01E-12	2.91	2.15–3.93
Type 2 diabetes	−0.84	0.0043	1.69E-05	0.43	0.29–0.64
Systolic blood pressure	0.03	0.0001	2.20E-07	1.03	1.02–1.05
Diastolic blood pressure	−0.05	0.0003	1.84E-05	0.95	0.93–0.98
Age	0.05	0.0001	1.63E-15	1.05	1.04–1.06
Weight	−0.01	3.94E-05	9.13E-07	0.99	0.98–0.99
Height	0.12	0.0001	2.78E-05	1.13	1.06–1.20
Sex	0.65	0.0044	0.00172	1.92	1.24–2.97
Benign neoplasm	−0.39	0.0029	0.00263	0.67	0.51–0.89
Pulmonary disease	0.54	0.0035	0.00061	1.72	1.24–2.38
Hypertension	0.62	0.0038	0.00056	1.86	1.28–2.72
Myelogenous neoplasm	−0.32	0.0032	0.02131	0.73	0.54–0.99

Controls chosen randomly from all non-AAA subjects (2,500 iterations, 500 cases, 2,000 controls), but weighted on sex and age (5 y classes) to match census population structure for the GHS catchment area. For outline of the study design, see Figure 1.

Age classes below 35 were consolidated into a single age class to avoid oversampling the sparse classes of [18, 20, 25] and [25, 30]. These classes are typically underrepresented in physician visits.

*Meta.Est is the meta-analysis estimate (random effects, variance weighted, aggregate) of the betas from bootstrap iterations, Meta SE is the standard error of this estimate.

^†^Two-sided *P*-value.

## Results

We identified 888 AAA cases from the GHS Department of Vascular Surgery. We also identified a pool of 10,523 patients without AAA from the Geisinger EMR who were consented into the MyCode® biobanking project with complete data for the variables of interest (Figure 1). Using 2010 census demographics, we standardized the control sample sets to match the demographics of the population residing in the GHS catchment area. After randomly generating 2,500 iterations of 500 cases and 2,000 controls each, we used meta-analysis and weighted the variables by how often they appeared in the 2,500 iterations and their significance (*P* value). The highest ranking variables were included in a second bootstrap analysis to obtain unbiased estimates (Table 2). Peripheral artery disease (PAD), smoking, coronary stenosis, systolic blood pressure, age, height (taller stature), male sex, pulmonary disease and hypertension were significantly associated with an increased risk for AAA. Type 2 diabetes mellitus (T2DM), diastolic blood pressure, weight, benign neoplasms and myelogenous neoplasms had a significantly negative association with AAA. Blood pressure remained in the model as diastolic and systolic measurements, as well as the diagnosis of hypertension.

The significant association between AAA and benign neoplasms was a novel finding. We compared all AAA cases with at least one benign neoplasm diagnosis (n = 365) to all controls with a benign neoplasm diagnosis (n = 5,419) (Table 3). Some individuals had more than one type of neoplasm. Benign neoplasm of the skin was the most common subtype in controls (73%), significantly more common than in cases (59%, *P* <0.001). Benign neoplasm of the digestive system was the most prevalent in cases (61%) as compared to controls (43%) and this difference was also significant (*P* <0.001). Benign neoplasm of the mouth/throat was only borderline significantly different between cases and controls, the remaining subtypes were not significantly different.

**Table 3:** **Comparison of subclasses of benign neoplasms between AAA cases and controls**

Type of neoplasm	Cases (n = 365) with neoplasm		Controls (n = 5419) with neoplasm		***P*** ^†^
	n	%	n	%	
Skin	216	59.18	3962	73.11	<0.001
Digestive	226	61.92	2317	42.76	<0.001
Benign Neoplasm NOS*	61	16.71	764	14.1	0.164
Lipoma	31	8.49	632	11.66	0.074
Hemangioma	12	3.29	248	4.58	0.296
Endocrine gland	22	6.03	211	3.89	0.053
Eye	10	2.74	106	1.96	0.33
Brain/nervous	7	1.92	104	1.92	0.98
Soft tissue	2	0.55	88	1.62	0.126
Mouth/throat	11	3.01	82	1.51	0.048
Respiratory	6	1.64	37	0.68	0.051
Urinary	2	0.55	21	0.39	0.654
Bone	0	0	10	0.18	0.969

*NOS = Not otherwise specified.

Cases and controls used for this analysis include those with at least one diagnosis of benign neoplasm. Controls which were selected at least 100 times in the bootstrap replication sets to provide a more closely matched comparison group.

^†^χ^2^or Fisher’s exact test where appropriate, *P*- values are 2-tailed.

## Discussion

**Figure 2:**
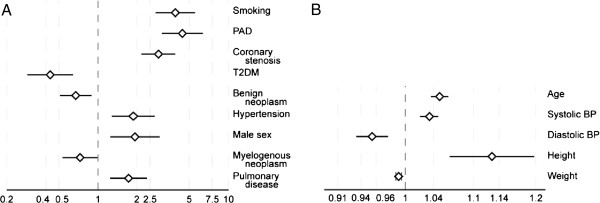
**Forest plot of risk factors for AAA identified using EMR data.** For scaling purposes, the data are separated into. **A**, discrete variables; and **B**, continuous variables. Odds ratios and 95% confidence intervals for the variables associated with AAA are shown. PAD, peripheral artery disease; T2DM, type 2 diabetes mellitus; BP, blood pressure.

This study demonstrated the feasibility of utilizing EMR data in a retrospective study for risk factor assessment of AAA, a complex disease. Previous studies have identified a number of risk factors for AAA including age, male sex, and smoking [28–38, 49–52] which were confirmed as important risk factors in the current study (Figure 2; Table 2). Age of the patient has also been significantly associated with survival based on repair type [53]. Strong and consistent evidence of an association of smoking with AAA warranted the inclusion of AAA in the Surgeon General’s report on The Heath Consequences of Smoking in 2004 [54]. Smoking also affects AAA expansion and rupture [36, 55, 56]. In addition, PAD [33, 34, 36, 56], coronary stenosis [31, 34, 36–38, 56], systolic blood pressure [32, 34, 41, 42], height [30, 31, 37, 41], pulmonary disease [39, 57], a diagnosis of hypertension [28, 30, 32, 34, 37, 41, 49, 55] and malignant neoplasms [58, 59] were all significantly associated with an increased AAA risk in this population. The negative association with T2DM replicated published AAA epidemiologic studies [23, 30, 34–36, 38, 55, 60]. Diabetes has also been associated with a decrease in growth of AAA [36]. A negative association was also found with weight. Height was found to be significantly associated with AAA, independent of body mass index (BMI), replicating published findings for AAA [30, 31, 36, 41]. We found a negative, but not statistically significant, association of BMI with AAA. In previous studies the association of BMI with AAA has been inconsistent, many studies have found a positive association with AAA [31, 38, 61], while others have found a negative association [36] or no association [32, 37, 41, 62].

We found a negative association of myelogenous (OR =0.73, *P* =0.021) neoplasms with AAA. An association of AAA and cancer has been reported in two studies when comparing AAA cases to patients with atherosclerotic occlusive disease (AOD) [58, 59]. The sample size in the first study was small, 69 AAA and 61 AOD cases [59]. The second study with a larger group, 298 AAA and 151 AOD patients also found an association of AAA with cancer, but it was not statistically significant when controlling for the confounders of age and smoking [58].

The negative association of benign neoplasms with AAA identified in the current study is intriguing. The most common type of neoplasm was of the skin, significantly more common in controls than cases. Neoplasms of the digestive system were more common in cases. The identified association is biologically plausible since two genes (*CDKN2BAS* and *DABP2*) with strong associations to AAA have roles in cell growth [63, 64]. Further research is necessary and may provide a clue to the molecular biology of AAA. Since the current study is cross-sectional, we cannot determine causation, but rather the results reveal correlations between incident AAA and various clinical variables.

The major strength of this study lies in the demonstration that EMR data collected as part of standard clinical care is suitable for retrospective epidemiologic analysis. This has profound implications for future use of EMR data for other risk analyses. The Medicare and Medicaid EHR Incentive Program provides incentives to eligible health care providers, and EMR data should become more readily available to a majority of researchers [2]. A limitation of EMRs, and this study, is that EMRs are designed for clinical utility rather than research purposes. Consequently, data entry errors such as missing observations, inconsistent entries, outliers or improbable values must be resolved prior to analysis. Approaches to using EMR data are being investigated by a number of groups including the NIH-funded electronic Medical Record and Genomics (eMERGE) Network [65, 66] and the Health Maintenance Organization Research Network (HMORN) Virtual Data Warehouse (VDW) project [67]. Despite gaps and inconsistencies in EMR data, the information available for our study was of sufficient quality to identify the major known risk factors for AAA. We focused on identifying risk factors for the incidence of AAA, not AAA progression, which likely has different risk factors.

As Geisinger is the primary health care provider for the population residing in Northeastern Pennsylvania, the EMR contains extensive medical history for all the patients in our study. The EMR allows us to mitigate several issues inherent in epidemiologic research, such as selection bias and unknown confounders. A major advantage of the EMR is that our study had much higher participation rates than traditional epidemiological studies, since data could be extracted from the EMR *post facto*. The proportion of elderly individuals in Central and Northeastern Pennsylvania is higher than the national average, increasing the number of AAA cases in the current study. A bias inherent to EMRs includes over-representation of sick participants and underrepresentation of the young, who tend to be healthy and less prone to seeking medical care. In our case, MyCode® recruits patients from primary care in addition to tertiary care, and the general health of the participants, therefore, is more representative of the general population. Generalizability of the results to other populations is unknown, although it is encouraging that all the known major risk factors found in previous studies in other populations were detected in our study. Previous studies have indicated that family history is a significant risk factor [30, 39, 51, 68, 69] but family history of AAA was not recorded in the Geisinger EMR.

## Conclusions

One of the goals of the study was to identify risk factors for AAA which could then be used to refine the eligibility criteria for AAA ultrasonography screening programs. The current screening guidelines have low sensitivity and specificity, and an improved risk prediction model would be of great public health benefit [38, 70]. Future work on AAA risk prediction models should include genotypes of genetic variants [63, 71–73] along with the recognized demographic and clinical variables. We would also like to study risk factors for progression and growth rates of AAAs. EMRs allow researchers to rapidly and inexpensively use clinical data to expand cohort size to derive better estimates for AAA as well as other complex diseases.

## Abbreviations

AAA: Abdominal aortic aneurysm
USPSTF: United States Preventative Services Task Force
GHS: Geisinger Health System
EMR: Electronic medical records
eMERGE: Electronic MEdical Records and Genomics
BP: Blood pressure
BMI: Body mass index
HbA1c: Glycosylated hemoglobin
MyCode®: Geisinger system-wide biobanking program of adult primary care patients
PAD: Peripheral artery disease
OR: Odds ratio
CI: Confidence interval
ICD-9: *International Classification of Diseases*, Ninth Revision
CPT: Current Procedural Terminology.
